# ¿Do we prescribe less clozapine to immigrant psychotic patients compared to non-immigrant psychotic patients?

**DOI:** 10.1192/j.eurpsy.2022.566

**Published:** 2022-09-01

**Authors:** A. Trabsa, A. Mané, A. Llimona González, L. Vargas, C. Muro, A. Moreno, B. Amann, V. Pérez-Solà

**Affiliations:** 1 Hospital del Mar (Consorci Mar Parc de Salut de Barcelona), Psychiatry, Barcelona, Spain; 2 Parc de Salut Mar, Institut De Neuropsiquiatria I Addiccions, Barcelona, Spain; 3 Hospital del Mar, Psychiatry, Barcelona, Spain

**Keywords:** migration psychiatry, psychopharmacology, Transcultural psychiatry, Psychosis

## Abstract

**Introduction:**

Clozapine, the first atypical antipsychotic, is a highly effective medication for patients with treatment-resistant schizophrenia. Robust evidence describes important risk for psychosis in immigrant population(2). Despite this, some studies suggest that immigrant patients are less treated and misdiagnosed due to cultural barriers(3,4). Clozapine and Electroconvulsive therapy tend to be less prescribed in immigrants(3). However, few studies assess differences in clozapine prescription between immigrants and non-immigrant psychotic inpatients.

**Objectives:**

To describe and compare clozapine prescription between psychotic patients and non-psychotic patients in a sample of Acute and Chronic inpatients.

**Methods:**

Patients who have presented, according to DSM-V criteria, one or more non-affective psychotic episodes, were recruited in Acute and Chronic inpatients units leading to a total sample of 198 patients. Immigrant condition was defined as “a person who comes to live permanently in a foreign country”. Demographic characteristics of patients, clinical data and main pharmacological treatment were recorded through a questionnaire. Comparative analysis was performed with IBM SPSS Statistics using Chi-Square Test and t-Student test.

**Results:**

From a total of 198patients clozapine was prescribed to 31(15,7%). From the total immigrant sample only 7,1% had prescribed clozapine compared to 24,2% from the locals(p<0.005). Significant differences in diagnosis associated to clozapine were found between both groups : Schizophrenia(57,1%immigrants, 57,1%locals), Schizoaffective disorder(14,3%immigrants, 41,7%locals) and Non specific psychosis (28,3%immigrants, 8,3%locals).

**Conclusions:**

According to our results, immigrant psychotic inpatients receive less clozapine prescription compared to non-immigrant psychotic patients. There results should be considered to study barriers for clozapine prescription in this population and offer a treatment based in equality.

**Disclosure:**

No significant relationships.

